# An atypical variant of congenital bronchoesophageal fistula associated with esophageal atresia and right main bronchial atresia and right lung hypoplasia in a preterm neonate: a case report

**DOI:** 10.1186/s13256-026-06250-1

**Published:** 2026-06-27

**Authors:** Anas Abdulkader, Youssef Al-Jallad, Samira M. Ghallab, Kusay Adwan, Usama Etman, Yasser Abdelhady Attia, Namrah Khalid, Raafat Bekhit, Omar A. Ghidan, Noof K. Binashikhbubkr, Nabil Shehata

**Affiliations:** 1 College of Medicine, AlMaarefa University, Riyadh, Saudi Arabia; 2 College of Medicine, Alfaisal University, Riyadh, Saudi Arabia; 3 Department of Neonatology, Saudi German Hospital, Riyadh, Saudi Arabia; 4 Department of Radiology, Saudi German Hospital, Riyadh, Saudi Arabia; 5 College of Medicine, Arab Academy for Science, Technology and Maritime Transport, El Alamein, Egypt; 6 College of Medicine and Health Science, Hadhramout University, Hadhramout, Yemen

**Keywords:** Communicating bronchopulmonary foregut malformation, CBPFM, Esophageal atresia, Right lung hypoplasia, Dextroposition, Pneumonectomy

## Abstract

**Background:**

Congenital bronchoesophageal fistula (BEF) is a rare communicating bronchopulmonary foregut malformation (CBPFM) characterized by an abnormal connection between the esophagus and a bronchial segment or lung tissue. While esophageal atresia (EA), with or without tracheoesophageal fistula, occurs in approximately 2–3 per 10,000 live births, EA associated with CBPFM/congenital BEF is far rarer, with no established population-based incidence and only several dozen reported CBPFM cases in the literature. Most congenital BEFs present in childhood or adulthood with chronic, nonspecific respiratory symptoms and recurrent infections, whereas neonatal presentations are typically linked to major associated anomalies such as EA. Importantly, complex neonatal constellations—particularly those combining EA with bronchial atresia and a severely hypoplastic, non-aerated lung—may represent an atypical CBPFM variant that does not fit neatly within traditional BEF classification schemes.

**Case presentation:**

We report a premature Arab female infant born at 32 weeks gestation who presented with respiratory distress, copious oral secretions, and radiographic features of EA with complete right hemithorax opacification and mediastinal shift to the right on a chest X-ray (CXR). After initial stabilization, a gastrostomy tube was inserted. Contrast studies via the gastrostomy tube and computed tomography angiography (CTA) revealed EA with a fistulous communication between the distal esophagus and right bronchial structures, along with a severely hypoplastic right lung. On day 33 of life, the patient underwent surgery. Intraoperative findings confirmed a fistula between the atretic right main bronchus and the lower esophagus, with a nonfunctioning right lung. Definitive EA repair and right pneumonectomy were performed. Histopathology revealed bronchial atresia involving the right main bronchus with right pulmonary hypoplasia. The postoperative course was complicated by pneumothorax, pneumomediastinum, sepsis, and persistent anastomotic leakage, and was managed conservatively with drainage, antimicrobial/antifungal therapy, and prolonged total parenteral nutrition. Serial contrast studies confirmed gradual leak resolution, and oral feeding was started by postoperative day 87. On follow-up, the infant initially maintained oxygen saturation and stable vital signs in room air without respiratory support; however, feeding advancement was complicated by recurrent vomiting and suspected delayed gastric emptying/gastroparesis. Despite combined parenteral and enteral nutrition and a trial of continuous orogastric tube feeding, the infant later developed severe septic shock requiring intravenous antibiotics and ventilatory support. The septic shock was refractory, and the patient died at 5 months of chronological age, corresponding to approximately 3 months corrected age.

**Conclusion:**

This case represents an atypical congenital BEF variant in which EA coexists with a distal esophageal fistula to an atretic right main bronchus and a severely hypoplastic, non-aerated right lung, highlighting a classification-challenging phenotype. It underscores the crucial role of early targeted imaging in neonates with suspected EA and unilateral lung hypoplasia/atresia. Advanced imaging enables accurate diagnosis and guides surgical planning, while the fatal follow-up course highlights the clinical vulnerability of these patients and the need for careful long-term multidisciplinary monitoring.

## Background

Congenital bronchoesophageal fistula (BEF) is an abnormal form of communication between the esophagus and a bronchial segment or lobe [1, 2]. These lesions are part of the communicating bronchopulmonary foregut malformations (CBPFMs), a rare group of anomalies where part of the respiratory tract communicates with the gastrointestinal tract, usually the esophagus.

Most congenital BEF cases present in childhood or adulthood with chronic, nonspecific respiratory symptoms and recurrent infections [1, 3]. When congenital BEF is associated with major anomalies such as esophageal atresia (EA), diagnosis occurs earlier.

Esophageal atresia (EA), with or without tracheoesophageal fistula, is a rare but well-recognized congenital anomaly, with population-based studies reporting an incidence of approximately 2–3 cases per 10,000 live births [4, 5]. In contrast, EA associated with communicating bronchopulmonary foregut malformation (CBPFM) or congenital bronchoesophageal fistula is exceptionally rare, and no robust population-based incidence has been established [6, 7]. A systematic review identified only 61 reported CBPFM cases worldwide since 1992, with Group I lesions, which are associated with EA/TEF, accounting for 27.9% of cases [7]. Therefore, EA with CBPFM represents a very uncommon subset of EA, described mainly through isolated case reports and small case series [6, 7].

This report describes a preterm neonate with congenital BEF, right lung hypoplasia, and long-gap EA, summarizing the clinical presentation, imaging, intraoperative findings, and surgical management of this complex anomaly.

## Case presentation

A premature Arab female infant born at 32 weeks’ gestation, with a birth weight of 1.6 kg, developed copious oral secretions and respiratory distress immediately after delivery. These findings were suggestive of esophageal atresia (EA). The infant was initially started on noninvasive ventilation (NIV) but rapidly developed respiratory acidosis, with a venous blood gas showing pH 7.10 and pCO₂ 68 mmHg, requiring urgent intubation and mechanical ventilation.

The infant was transferred to our hospital on day 9 of life as a suspected case of EA with possible right lung collapse. On examination after transfer, the patient was intubated and mechanically ventilated, with stable oxygen saturation and no signs of acute clinical deterioration. The infant was not dysmorphic, and breath sounds were markedly diminished over the right lung. Abdominal examination and abdominal ultrasound were normal. Heart sounds were shifted to the right, but no murmurs were present.

**Fig. 1:**
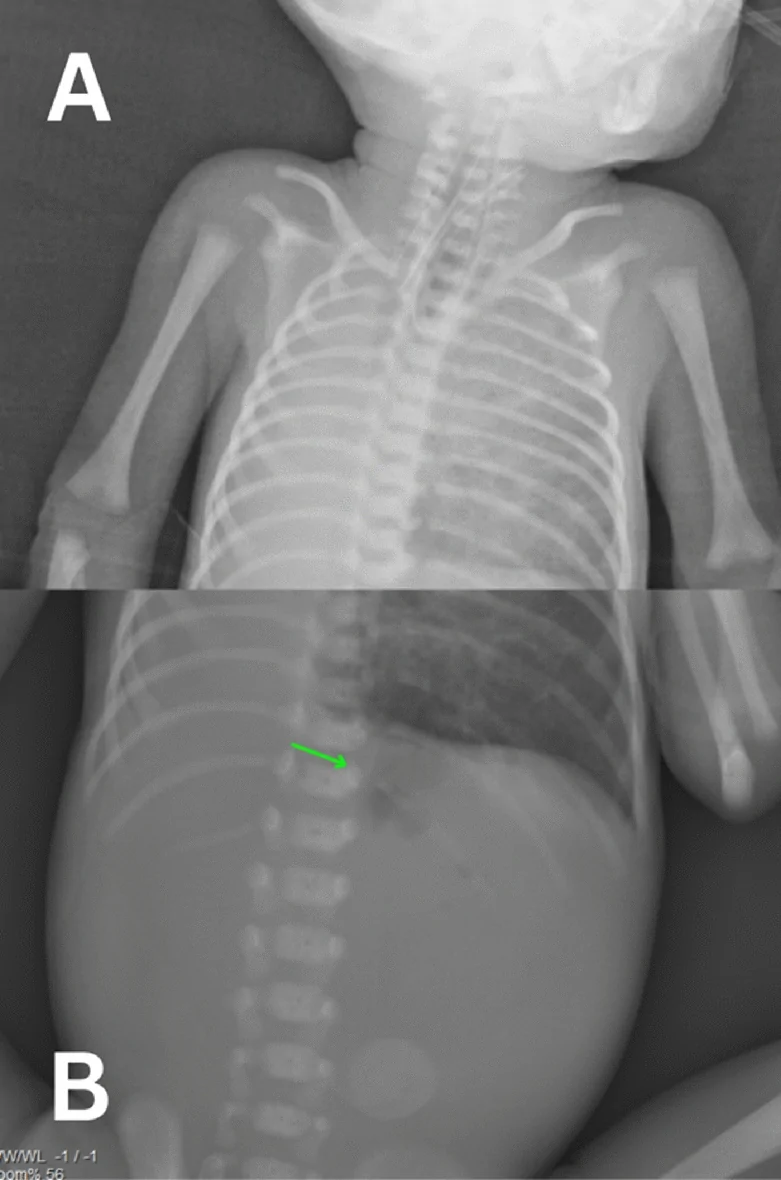
Initial chest radiography at admission (9th day of life). Panel **A** Plain radiograph demonstrating several critical findings. The endotracheal tube (ETT) is located in situ, whereas the orogastric tube (OGT) is appropriately positioned within the upper mediastinum. There was a complete right-sided white hemithorax with compensatory hyperinflation of the left lung. The cardiac silhouette and mediastinum are shifted to the right. The hyperinflated left lung exhibited diffuse ground-glass and reticulated opacities. Panel **B** The abdominal portion of the same radiograph demonstrates a normal bowel gas pattern (green arrow)

Chest X-ray (CXR) revealed the orogastric tube (OGT) coiled in the upper mediastinum, right hemithorax white-out, mediastinal shift to the right, and hyperinflation of the left lung with diffuse ground-glass and reticulated opacities (Fig. 1).

Given these findings, a full anomaly work-up, including follow-up imaging and echocardiography, was performed. Echocardiography revealed dextroposition, severe right pulmonary artery hypoplasia measuring 2 mm with flow reversal, minimal forward flow to the right lung, a normal left pulmonary artery measuring 4.5 mm, and minimal flow in the right pulmonary veins. A small patent ductus arteriosus (PDA) with a left-to-right shunt and a patent foramen ovale (PFO) with a left-to-right shunt were also noted. Cardiac anatomy and function were otherwise normal, with no pulmonary hypertension, clots, or vegetation.

The genetic work-up included karyotyping, which revealed 46,XX,9qh+. Whole-exome sequencing was recommended but was declined by the parents.

**Fig. 2:**
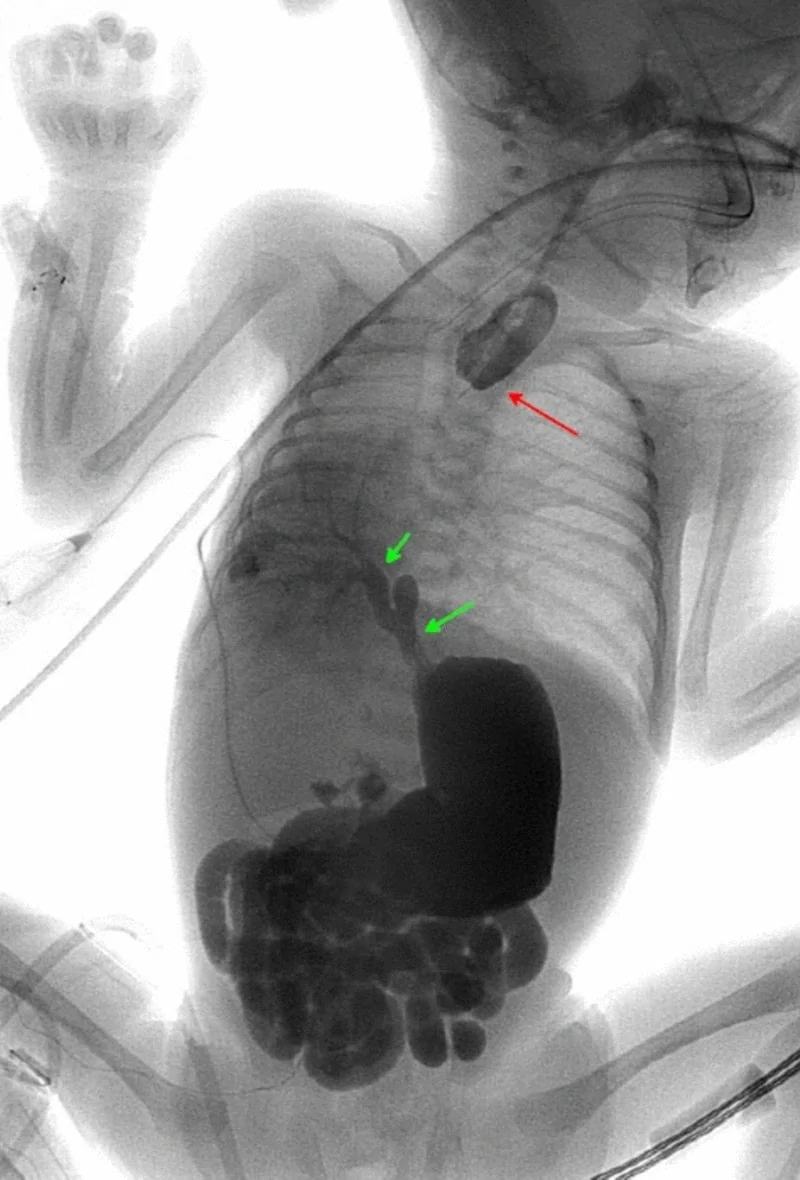
Preoperative Gastrografin study (aged 29 days). An anterior–posterior radiograph demonstrating contrast administration through a gastrostomy tube and a nasogastric tube. The nasogastric tube terminates in a dilated, blind-ended upper esophageal pouch in the upper thoracic region, consistent with esophageal atresia (EA). Following Gastrografin administration, an abnormal tract is opacified. This tract appears to communicate with an atretic/hypoplastic right lung, where the contrast outlines a branching pattern resembling the bronchial tree. These findings are highly suggestive of congenital EA with a bronchoesophageal fistula, likely associated with pulmonary aplasia/hypoplasia of the right lung. The radiological features support a complex congenital foregut malformation and demonstrate the utility of contrast studies for delineating aberrant fistulous communications in the neonatal period

Given the patient’s condition, a gastrostomy tube was inserted on day 12 of life. A Gastrografin contrast study revealed findings highly suggestive of congenital EA with a bronchoesophageal fistula, likely associated with pulmonary aplasia/hypoplasia of the right lung (Fig. 2).

**Fig. 3:**
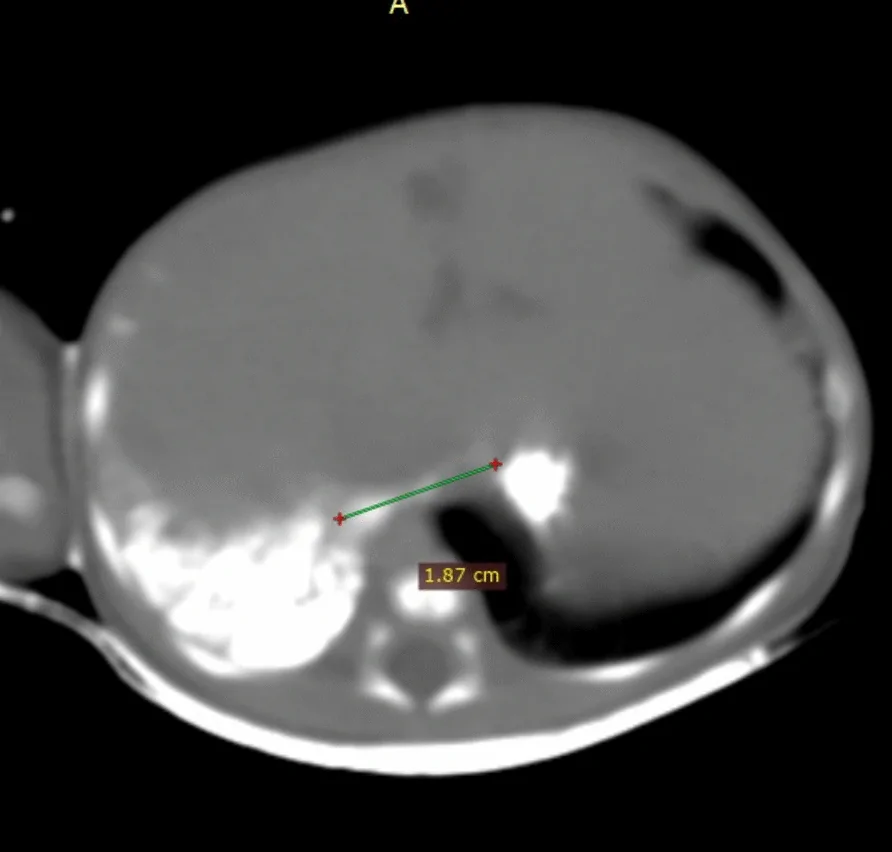
Axial computed tomography with Gastrografin contrast. Axial computed tomography following Gastrografin administration through the gastrostomy tube confirms a fistulous connection between the distal esophagus and an atretic right lung (arrow), with alveolar opacification due to contrast filling

The full extent of the malformation was revealed by computed tomography (CT) of the chest and abdomen (Fig. 3). Combined computed tomography angiography (CTA) was performed with intravenous contrast and contrast administration through the gastrostomy tube. The abdominal structures were normal. Contrast flow from the gastrostomy tube entered a linear structure communicating with the right lung bronchi and hypoplastic parenchyma, confirming an abnormal connection between the gastroesophageal junction and the hypoplastic right lung.

Multislice CT pulmonary angiography showed rightward displacement of the heart and reduced diameter of the right main pulmonary artery, consistent with right pulmonary artery hypoplasia and hypoplastic right pulmonary veins. No anomalous pulmonary venous drainage was observed. Bronchoscopy was not performed, as the fistulous anatomy and pulmonary vascular findings were adequately delineated by contrast studies and CTA.

On day 33 of life, surgical correction was performed via right thoracotomy. Intraoperatively, a fistulous tract between the distal esophagus and a severely hypoplastic right lung was identified, ligated, and divided. Since the right lung was non-aerated and irreversibly hypoplastic, right pneumonectomy was performed. Esophageal continuity was restored with proximal and distal end-to-end anastomosis. A transanastomotic nasogastric tube (NGT) was left for decompression and enteral access. No chest tube was inserted.

Histopathology of the excised right lung confirmed bronchial atresia of the right main bronchus, with a hypoplastic, non-aerated, fibrotic lung and no evidence of malignancy or specific infection. An esophageal lung variant was considered but excluded on the basis of CTA and intraoperative findings.

Postoperative complications included air leakage, surgical emphysema, pneumomediastinum, and pneumothorax, requiring intercostal tube (ICT) insertion. The infant also developed neonatal sepsis and was treated with appropriate antibiotics.

**Fig. 4:**
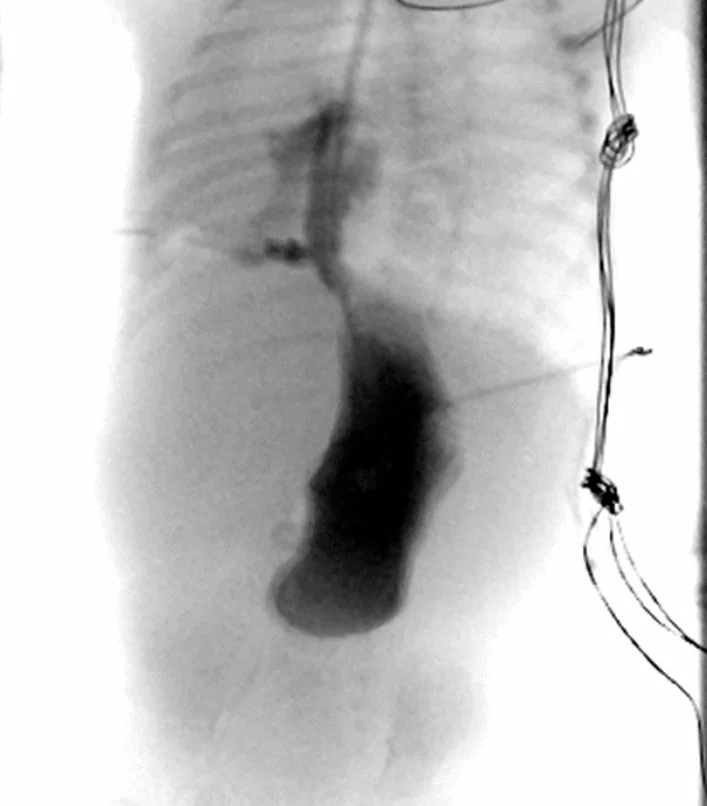
Postoperative gastrografin study. This follow-up study shows a contrast tract with extravasation, consistent with an anastomotic leak

**Fig. 5:**
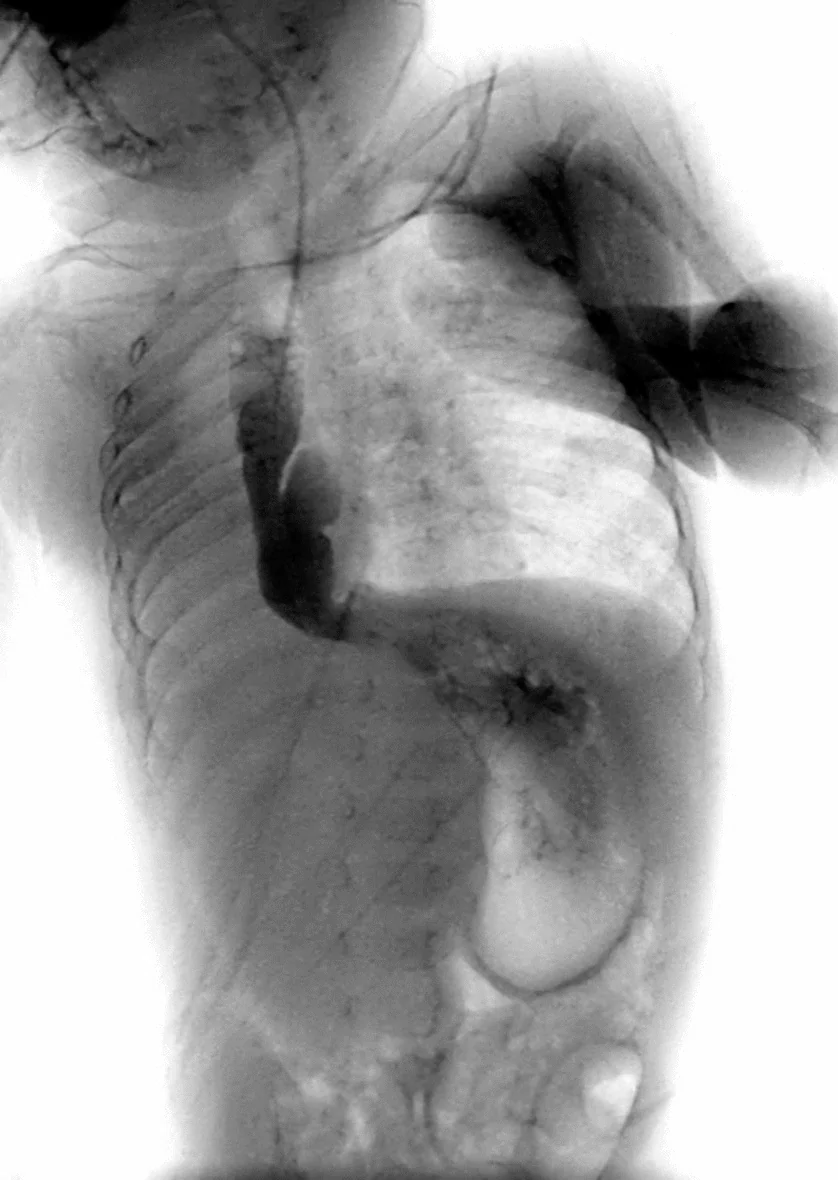
Final postoperative Gastrografin study. Postoperative Gastrografin administration through a nasogastric tube shows restoration of esophageal continuity with no evidence of residual or recurrent fistula

On postoperative day 12, a Gastrografin swallow revealed a large anastomotic leak with significant extravasation into the post-pneumonectomy space. From postoperative days 13 to 50, serial follow-up swallow studies showed progressive reduction and eventual resolution of the leak, with documented gastroesophageal reflux (Fig. 4). Management throughout this period was conservative with nil per os (NPO), total parenteral nutrition (TPN), and intravenous antibiotics/antifungals. During this interval, the infant was successfully extubated and maintained comfortable spontaneous breathing in room air. The gastrostomy tube was inadvertently dislodged, with spontaneous closure of the opening (Fig. 5). On postoperative day 87, the infant had a stable examination with a healed anastomosis, and oral feeds with anti-regurgitation formula were initiated with reflux precautions.

On follow-up, the infant initially maintained adequate oxygen saturation and stable vital signs in room air without respiratory support. However, feeding advancement was poorly tolerated, with recurrent vomiting whenever enteral feeds approached approximately half of the target volume. Mechanical causes of obstruction were excluded. Following contrast evaluation, delayed gastric emptying/gastroparesis was suspected. Therefore, a trial of continuous orogastric tube feeding was initiated, with a plan to proceed to nasojejunal tube feeding if feeding intolerance persisted. During this period, the patient received combined parenteral and enteral nutrition. When feeds were again advanced beyond approximately half of the target volume, the infant developed clinical signs of sepsis and progressed to severe septic shock. A septic work-up was performed, and intravenous antibiotics and ventilatory support were initiated. Despite intensive management, the septic shock was refractory, and the patient died at 5 months of chronological age, corresponding to approximately 3 months corrected age.

**Fig. 6:**
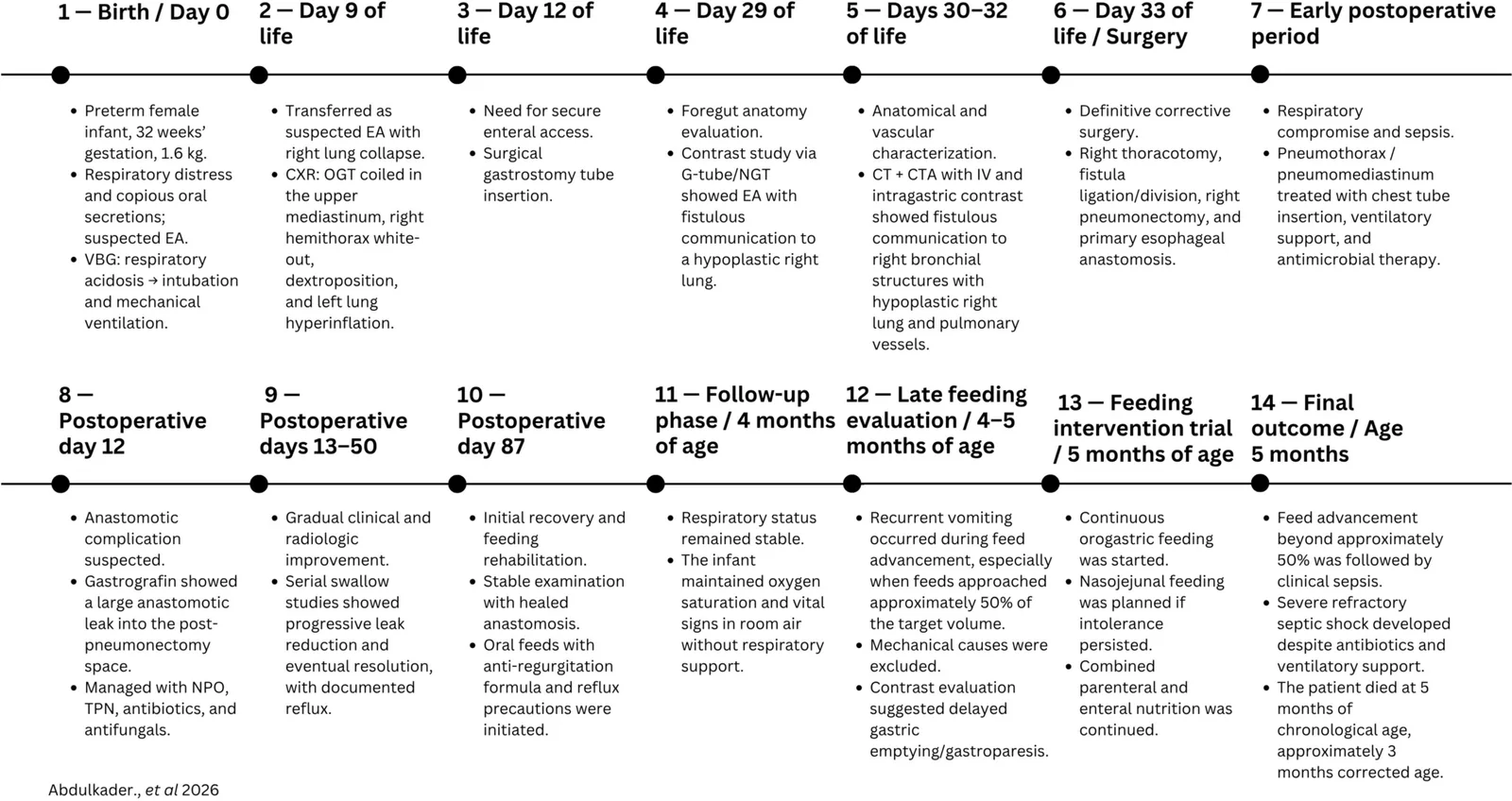
Clinical timeline of the patient’s diagnostic, surgical, and follow-up course. The timeline summarizes key events from birth to final follow-up, including initial respiratory presentation, diagnostic imaging, surgical repair, postoperative anastomotic leak, feeding intolerance, suspected delayed gastric emptying/gastroparesis, septic shock, and final outcome. *EA* esophageal atresia, *VBG* venous blood gas, *CXR* chest X-ray, *OGT* orogastric tube, *G-tube* gastrostomy tube, *NGT* nasogastric tube, *CT* computed tomography, *CTA* computed tomography angiography, *IV* intravenous, *NPO* nil per os, *TPN* total parenteral nutrition

A visual summary of the clinical course is presented in Fig. 6.

## Discussion

Congenital BEF is a rare foregut–respiratory malformation in which a bronchus or lung segment abnormally communicates with the esophagus. It can closely mimic more common neonatal entities, such as EA with or without tracheoesophageal fistula (TEF), unilateral lung collapse, or pulmonary hypoplasia. The available literature is limited to case reports and small series, which hampers the development of standardized diagnostic and management strategies. As a result, misdiagnosis and delayed recognition are frequent, predisposing infants to recurrent lower respiratory infections, persistent respiratory distress, and delayed surgical repair.

Congenital BEF is thought to result from abnormal separation of the primitive foregut into the dorsal esophagus and ventral tracheobronchial tree during weeks 4–5 of gestation. Proposed mechanisms include defects in the tracheobronchial mesoderm and misdirection of a lung bud toward the esophagus, leading to separation from the main pulmonary primordium. The most common anatomical subtype is a fistulous connection between the middle third of the esophagus and the right lower lobe bronchus in a normally developed bronchial segment [1, 8–11].

The Braimbridge and Keith classification describes congenital bronchoesophageal fistulas according to the anatomical configuration between the esophagus and the bronchial system, including connections to either normally aerated bronchial segments or sequestered lung tissue, whereas the Srikanth system places these lesions within the broader spectrum of CBPFM and stratifies them by their association with esophageal atresia/tracheoesophageal fistula and by the extent of pulmonary involvement [1, 11–13]. Our patient’s anomaly clearly falls within the CBPFM spectrum but does not conform neatly to any of the four Braimbridge types because the right main bronchus was atretic and the right lung was severely hypoplastic and non-aerated rather than a normally ventilated segment or a discrete sequestered lobe. Given the coexistence of EA and a fistulous communication between the distal esophagus and a severely hypoplastic right lung, this lesion is best regarded as an atypical form of congenital BEF/CBPFM characterized by right main bronchial atresia and unilateral right lung hypoplasia.

What makes this case particularly rare is not only the presence of congenital BEF, but the unusual combination of esophageal atresia, a distal esophageal communication to an atretic right main bronchus, and a severely hypoplastic, nonfunctional right lung. Unlike more typical congenital BEF cases, which usually involve a fistulous connection to a normally developed bronchial segment or sequestered lung tissue, this anatomical constellation represents a classification-challenging CBPFM variant. This distinction is clinically important because such cases may be misclassified preoperatively as standard EA/TEF, unilateral lung collapse, pulmonary agenesis, or isolated pulmonary hypoplasia.

Congenital BEF and CBPFM are often associated with additional thoracic and systemic malformations. Reported abnormalities include bronchiectasis, pulmonary sequestration, hybrid lesions, bronchial atresia, lobar emphysema, congenital heart defects, and gastrointestinal anomalies such as EA/TEF, often within the Vertebral, Anal, Cardiac, Tracheoesophageal, Renal, and Limb anomalies (VACTERL) association [6, 7, 12–19]. These associations highlight the need for systematic screening for other malformations in affected neonates, including detailed cardiopulmonary and gastrointestinal assessments.

In our patient, karyotyping revealed a female genotype, 46,XX,9qh+, which is considered a benign pericentromeric heterochromatin polymorphism [20–22]. No pathogenic chromosomal abnormality was identified, and in keeping with the current literature, no single causative gene or recurrent chromosomal rearrangement has yet been consistently implicated in CBPFM.

Congenital BEF in children is often diagnosed late or missed because symptoms may be subtle and nonspecific. Recurrent cough, choking with feeds, and repeated pneumonias are frequently attributed to more common conditions, such as asthma, recurrent bronchitis, or gastroesophageal reflux disease, leading to inappropriate or prolonged therapy before the fistula is recognized [6, 23–25]. For example, Alemu et al. described a 10-year-old girl treated for “refractory asthma” and recurrent pneumonia until a contrast esophagogram revealed an isolated BEF [26]. On the other hand, diagnosis may occur incidentally in the operating room when the anatomy does not match a presumed EA/TEF; in one neonate, intraoperative bronchoscopy revealed an esophago-bronchial communication, prompting a change in surgical strategy [27]. Similar diagnostic pitfalls have been reported across the CBPFM spectrum, especially in Group I, where up to 82.4% of EA/TEF-associated CBPFM cases were initially misclassified as isolated EA/TEF [7, 28]. Zarei et al. described a 3-month-old infant with a right esophageal lung initially labeled as isolated pulmonary hypoplasia and suspected TEF; only targeted contrast esophagography and chest CT revealed an esophago-bronchial connection aerating a hypoplastic right lung, showing how unilateral “white-out” and recurrent postprandial respiratory distress can be misattributed to more common diagnoses in infancy [29].

Our infant exemplified these diagnostic challenges. The combination of suspected EA, right-sided white hemithorax, mediastinal shift, and dextroposition could easily have been misinterpreted as isolated EA with right lung collapse. However, a structured anomaly work-up refined the diagnosis. Echocardiography demonstrated dextroposition with severe hypoplasia of the right pulmonary artery and veins but otherwise normal intracardiac anatomy. Gastrografin study and CTA with intravascular and intragastric contrast demonstrated an esophago-bronchial communication supplying a severely hypoplastic, non-aerated right lung. The absence of anomalous right pulmonary venous drainage to the inferior vena cava and the lack of systemic arterial supply effectively excluded Scimitar syndrome, while histopathology confirmed right main bronchial atresia with marked pulmonary hypoplasia, ruling out alternative causes of unilateral neonatal lung opacity, such as uncomplicated atelectasis or primary lung agenesis.

In neonates with suspected EA and persistent unilateral hemithorax opacification, mediastinal shift, or suspected lung hypoplasia, a structured preoperative diagnostic approach is essential for surgical planning. This approach should include targeted contrast evaluation through appropriate access, CTA with vascular mapping, and echocardiography to assess pulmonary vasculature and associated cardiac anomalies. Bronchoscopy may also be considered when feasible to delineate airway anatomy, particularly when the relationship between the tracheobronchial tree, esophagus, and suspected fistulous tract remains uncertain. Such an algorithm can prevent misclassification as standard EA/TEF and can help guide operative strategy, including the need for fistula ligation, esophageal repair, lung resection, or pneumonectomy.

In the present case, bronchoscopy was not performed because the fistulous anatomy, pulmonary vascular status, and relationship of the hypoplastic right lung to the foregut were sufficiently delineated by contrast studies and CTA, and were subsequently confirmed by intraoperative findings and histopathology. Therefore, in selected cases, bronchoscopy may serve as a useful adjunct, particularly when airway anatomy remains uncertain; however, it should not be considered mandatory when cross-sectional and contrast imaging provide adequate anatomical definition.

An esophageal lung variant was considered but deemed unlikely, as CTA and intraoperative findings revealed a rudimentary right main bronchus from the trachea and a separate fistulous communication between the distal esophagus and the hypoplastic right lung. Histopathology revealed fibrotic and hypoplastic bronchial structures without a developed airway lumen, confirming right main bronchial atresia. The adjacent lung was atelectatic, with alveolar underdevelopment and interstitial fibrosis. These findings support a diagnosis of congenital bronchoesophageal fistula with right main bronchial atresia and pulmonary hypoplasia, rather than esophageal lung, in which the main bronchus originates from the esophagus and no tracheal right main bronchus is present.

Surgical excision remains the treatment of choice for congenital BEF in children and the broader CBPFM spectrum. Definitive management usually involves a thoracic approach tailored to the fistula location, with tract division, separate closure of esophageal and bronchial defects, and, when needed, reinforcement with muscle or pleural flaps. This may be combined with resection of nonfunctional lung segments or lobes [8, 30–33]. Minimally invasive thoracoscopic techniques are increasingly used as alternatives to open thoracotomy in favorable cases. For example, thoracoscopic left lower lobectomy with *en bloc* excision of the fistulous tract was performed at 5 years of age, confirming the feasibility of video-assisted thoracoscopic surgery (VATS) for selected pediatric CBPFM patients [34]. In our case, however, the neonatal age, low body weight, complex anatomy, long-gap EA, and severely hypoplastic nonfunctional right lung supported an open approach with right thoracotomy, fistula division, esophageal repair, and right pneumonectomy.

Endoscopic, nonsurgical interventions such as clipping, suturing, stenting, adhesive injection, or chemocauterization have been increasingly used in common airway fistulas, especially in children with TEF, including recurrent or H-type TEF [35–37]. In contrast, evidence for congenital BEF or other respiratory tract fistulas in children is minimal, and these techniques are not standard of care. Esophageal stenting in pediatric airway–esophageal fistulas often serves only as a bridge to surgery, with high rates of migration and risks of severe complications, including fatal outcomes [38]. In infants with tuberculosis-associated BEF, esophageal stents frequently cause tracheal compression requiring removal, and fibrin glue often fails, requiring surgical salvage [39]. Adult data show a low success rate of 45.5% for endoscopic closure of benign acquired fistulas, with over-the-scope clips failing in most cases [40]. Successful stenting for malignant BEF has been reported, but only as palliation in terminal disease, not as curative treatment [41]. Therefore, owing to anatomical challenges in neonates and the risks of airway compression or device migration, surgical excision remains the standard of care. Endoscopic strategies should be considered investigational and reserved for cases where surgery is not feasible.

Preoperative optimization, including infection control, airway management, and nutritional support, is critical to minimize perioperative risk [31]. In complex neonatal CBPFM, optimization must also account for prematurity, limited pulmonary reserve, associated vascular hypoplasia, and the anticipated need for prolonged postoperative nutritional support. In such cases, multidisciplinary planning involving neonatology, pediatric surgery, radiology, cardiology, nutrition, and intensive care is essential.

Although congenital BEF may have favorable outcomes after early recognition and surgical repair, prognosis in complex neonatal variants depends heavily on associated anomalies, pulmonary reserve, postoperative complications, feeding tolerance, and infectious morbidity. In the present case, initial anatomical repair and eventual leak resolution were achieved; however, the subsequent clinical course was complicated by feeding intolerance, suspected delayed gastric emptying/gastroparesis, recurrent sepsis, and ultimately refractory septic shock. This outcome highlights that successful anatomical correction does not necessarily predict an uncomplicated long-term clinical course in premature neonates with complex CBPFM.

Anastomotic leakage is a well-recognized early complication following esophageal repair, with reported rates of 5–15% and higher rates in complex cases such as long-gap or staged repairs [42–44]. These data broadly reflect the intrinsic risk of leakage across congenital foregut malformations, including rare conditions such as congenital BEF and CBPFM. In the present case, a persistent anastomotic leak was managed conservatively with NPO, broad-spectrum antibiotics, antifungal therapy, and parenteral nutrition until spontaneous resolution, thereby avoiding high-risk surgical reintervention. While conservative management is commonly practiced, detailed case-level reports in this specific context are limited. This experience highlights that prolonged nonoperative management of anastomotic leaks can be successful in selected neonates, provided that adequate systemic support is available. It also underscores the importance of vigilant postoperative monitoring for both airway and anastomotic complications, even after technically successful anatomical repair.

Feeding intolerance was an important late clinical issue in this case. Despite radiological healing of the anastomosis and initial respiratory stability in room air, enteral feeding advancement was repeatedly limited by vomiting, particularly when feeds approached approximately half of the target volume. Mechanical obstruction was excluded, and delayed gastric emptying/gastroparesis was suspected after contrast evaluation. A trial of continuous orogastric tube feeding was initiated, with a plan for nasojejunal tube feeding if intolerance persisted. This emphasizes the need to monitor not only respiratory and anastomotic outcomes, but also gastrointestinal motility, reflux, nutritional tolerance, and the risks associated with prolonged combined parenteral and enteral nutrition after complex EA/CBPFM repair.

In our case, right pneumonectomy was performed in a 1.6-kg neonate with a severely hypoplastic, nonfunctional lung. This decision illustrates the complex balance between removing a chronically noncontributory, infection-prone lung and preserving maximal respiratory reserve for future growth. Although the resected lung was nonfunctional, the long-term impact of pneumonectomy in early infancy on contralateral lung growth, chest wall development, exercise tolerance, and quality of life remains incompletely defined. These considerations highlight the importance of multidisciplinary decision-making, careful preoperative counseling of parents, and structured long-term respiratory follow-up.

The fatal outcome in this patient was related to refractory septic shock following a prolonged and complicated postoperative course. While a direct causal relationship with the anatomical repair cannot be established from a single case, the outcome underscores the vulnerability of premature infants with complex foregut–pulmonary malformations, single functional lung physiology, prolonged nutritional support, feeding intolerance, and recurrent infectious complications. Similarly, the fatal postoperative course reported in an infant who died from respiratory insufficiency after right pneumonectomy for a nonfunctional esophageal lung highlights the minimal respiratory reserve and airway vulnerability in neonates with a single functional lung [29]. Together, these experiences emphasize the need for meticulous postoperative monitoring, infection surveillance, feeding assessment, respiratory follow-up, and rescue planning in complex neonatal CBPFM cases.

This report has several limitations. First, it describes a single patient, which limits generalizability. Second, the rarity and anatomical complexity of the lesion make direct comparison with previously published cases difficult. Third, although the diagnosis was supported by contrast imaging, CTA, intraoperative findings, and histopathology, standardized diagnostic pathways for this specific phenotype remain lacking. Fourth, long-term functional respiratory and feeding outcomes could not be assessed because the infant died at 5 months of chronological age. Finally, because evidence for this condition is largely limited to case reports and small series, conclusions regarding optimal diagnostic sequencing, surgical timing, feeding management, and long-term prognosis should be interpreted cautiously. Larger multicenter reports are needed to better define diagnostic algorithms, operative strategies, postoperative care, and outcomes for rare CBPFM variants associated with EA and unilateral lung hypoplasia.

## Conclusion

This case highlights an atypical congenital BEF/CBPFM variant in which EA coexisted with a distal esophageal fistula to an atretic right main bronchus and a severely hypoplastic, nonfunctional right lung. The case emphasizes that persistent unilateral hemithorax opacification in a neonate with suspected EA should prompt early structured diagnostic evaluation using contrast studies, CTA with vascular mapping, and echocardiography to guide surgical planning. Although anatomical repair and leak resolution were achieved, the subsequent fatal course due to refractory septic shock highlights the vulnerability of premature neonates with complex foregut–pulmonary malformations and the need for careful multidisciplinary postoperative monitoring.

## Data Availability

No datasets were generated or analyzed during the current study.

## Abbreviations

BEF: Bronchoesophageal fistula
CBPFM: Communicating bronchopulmonary foregut malformation
CT: Computed tomography
CTA: Computed tomography angiography
CXR: Chest X-ray
EA: Esophageal atresia
ETT: Endotracheal tube
G-tube: Gastrostomy tube
ICT: Intercostal tube
IV: Intravenous
NGT: Nasogastric tube
NIV: Noninvasive ventilation
NPO: Nil per os (nothing by mouth)
OGT: Orogastric tube
OTSC: Over-the-scope clip
PA: Pulmonary artery
PDA: Patent ductus arteriosus
PFO: Patent foramen ovale
TEF: Tracheoesophageal fistula
TPN: Total parenteral nutrition
VACTERL: Vertebral, Anal, Cardiac, Tracheoesophageal, Renal, and Limb anomalies
VATS: Video-assisted thoracoscopic surgery

## References

[1] Braimbridge MV, Keith HI. Oesophago-bronchial fistula in the adult. Thorax. 1965;20:226–33. 14292429 10.1136/thx.20.3.226 PMC1018926

[2] Duprez A, Wittek F, Dumont A. Acquired and congenital oesophago-bronchial fistulas. Thorax. 1956;11:249–56. 13371575 10.1136/thx.11.3.249 PMC1019348

[3] Azoulay D, Regnard JF, Magdeleinat P, Diamond T, Rojas-Miranda A, Levasseur P, *et al*. Congenital respiratory-esophageal fistula in the adult. J Thorac Cardiovasc Surg. 1992;104:381–4. 1495299

[4] Oddsberg J, Lu Y, Lagergren J. Aspects of esophageal atresia in a population-based setting: incidence, mortality, and cancer risk. Pediatr Surg Int. 2012;28:249–57. 10.1007/s00383-011-3014-1. 22020495

[5] Sfeir R, Michaud L, Salleron J, Gottrand F. Epidemiology of esophageal atresia. Dis Esophagus. 2013;26(4):354–5. 10.1111/dote.12051. 23679022

[6] Ren H, Duan L, Zhao B, Wu X, Zhang H, Liu C. Diagnosis and treatment of communicating bronchopulmonary foregut malformation. Medicine. 2017;96: e6307. 10.1097/MD.0000000000006307. 28296740 PMC5369895

[7] Yang G, Chen L, Xu C, Yuan M, Li Y. Congenital bronchopulmonary foregut malformation: systematic review of the literature. BMC Pediatr. 2019;19:305. 10.1186/s12887-019-1686-1. 31477056 PMC6721191

[8] Risher WH, Arensman RM, Ochsner JL. Congenital bronchoesophageal fistula. Ann Thorac Surg. 1990;49:500–5. 2178573 10.1016/0003-4975(90)90274-a

[9] Kim JH, Park K-H, Sung NSW, Rho NJR. Congenital bronchoesophageal fistulas in adult patients. Ann Thorac Surg. 1995;60:151–5. 7598578 10.1016/s0003-4975(95)00326-6

[10] Zhang B-S. Congenital bronchoesophageal fistula in adults. World J Gastroenterol. 2011;17:1358. 21455337 10.3748/wjg.v17.i10.1358 PMC3068273

[11] Taira N, Kawasaki H, Atsumi E, Furugen T, Ichi T, Kushi K, *et al*. A rare case of congenital bronchoesophageal fistula in an adult. Int J Surg Case Rep. 2017;36:182–4. 28442319 10.1016/j.ijscr.2017.03.029 PMC5985245

[12] Kato H, Yoshikawa M, Saito T, Fukuchi M, Kato R, Kuwano H. Congenital Bronchoesophageal Fistula with Crohn’s Disease in an Adult: Report of a Case. Surg Today. 2001;31:446–9. 11381511 10.1007/s005950170138

[13] Srikanth MS, Ford EG, Stanley P, Mahour GH. Communicating bronchopulmonary foregut malformations: Classification and embryogenesis. J Pediatr Surg. 1992;27:732–6. 1501033 10.1016/s0022-3468(05)80103-4

[14] Imai Y, Mark EJ. Cystic adenomatoid change is common to various forms of cystic lung diseases of children. Arch Pathol Lab Med. 2002;126:934–40. 12171491 10.5858/2002-126-0934-CACICT

[15] Hermelijn SM, Zwartjes RR, Tiddens HAWM, Otter SCMC-D, Reiss IKM, Wijnen RMH, et al. Associated Anomalies in Congenital Lung Abnormalities: A 20-Year experience. Neonatology. 2020;117:697–703. 10.1159/000509426 32841951

[16] Pop D, Venissac N, Perrin C, Leo F, Padovani B, Mouroux J. Extralobar sequestration with anomalous pulmonary artery return or patent ductus arteriosus? J Thorac Cardiovasc Surg. 2005;130:903–4. 16153956 10.1016/j.jtcvs.2005.02.042

[17] Heithoff K, Sane S, Williams H, Jarvis C, Carter J, Kane P, *et al*. Bronchopulmonary foregut malformations. A unifying etiological concept. Am J Roentgenol. 1976;126:46–55. 175683 10.2214/ajr.126.1.46

[18] Leithiser R, Capitanio M, Macpherson R, Wood B. “Communicating” bronchopulmonary foregut malformations. Am J Roentgenol. 1986;146:227–31. 3484567 10.2214/ajr.146.2.227

[19] Brosens E, Felix JF, Munck AB-D, De Jong EM, Lodder EM, Swagemakers S, et al. Histological, immunohistochemical and transcriptomic characterization of human tracheoesophageal fistulas. PLoS ONE. 2020;15:e0242167. 10.1371/journal.pone.0242167 PMC7671559 33201890

[20] Madon PF, Athalye AS, Parikh FR. Polymorphic variants on chromosomes probably play a significant role in infertility. Reprod Biomed Online. 2005;11:726–32. 16417737 10.1016/s1472-6483(10)61691-4

[21] Kosyakova N, Grigorian A, Liehr T, Manvelyan M, Simonyan I, Mkrtchyan H, *et al*. Heteromorphic variants of chromosome 9. Mol Cytogenet. 2013;6:14. 23547710 10.1186/1755-8166-6-14 PMC3626942

[22] Mottola F, Santonastaso M, Ronga V, Finelli R, Rocco L. Polymorphic rearrangements of human chromosome 9 and male infertility: New evidence and impact on spermatogenesis. Biomolecules. 2023;13:729. 37238599 10.3390/biom13050729 PMC10216066

[23] LaSalle AJ, Andrassy RJ, Steeg KV, Ratner I. Congenital tracheoesophageal fistula without esophageal atresia. J Thorac Cardiovasc Surg. 1979;78:583–8. 480968

[24] Helmsworth JA, Pryles CV. Congenital tracheo-esophageal fistula without esophageal atresia. J Pediatr. 1951;38:610–7. 14832717 10.1016/s0022-3476(51)80303-2

[25] Wang Z-M, Zhang S-C, Teng X. Esophageal diverticulum serves as a unique cause of bronchoesophageal fistula in children. Medicine. 2017;96: e9492. 29390593 10.1097/MD.0000000000009492 PMC5758295

[26] Alemu S, Abera G, Kasaye A, Muhammed H, Demissie WR. Congenital broncho-esophageal fistula; presenting as recurrent pneumonia in a 10-years-old child: a case report. Int J Surg Case Rep. 2025;137: 112116. 41541120 10.1016/j.ijscr.2025.112116 PMC12596946

[27] Izzo S, Lo C, Stohl S. Case report of neonatal bronchoesophageal fistula with esophageal atresia. A&A Practice. 2025;19: e02081. 41128467 10.1213/XAA.0000000000002081

[28] He Q-M, Xiao S-J, Zhu X-C, Xiao W-Q, Wang Z, Zhong W, *et al*. Communicating bronchopulmonary foregut malformation type IA: radiologic anatomy and clinical dilemmas. Surg Radiol Anat. 2015;37:1251–6. 26077024 10.1007/s00276-015-1504-x

[29] Zarei E, Rakhshankhah N, Nejad NH, Eshghi A. Esophageal lung misdiagnosed as tracheoesophageal fistula: a case report. Radiology Case Reports. 2023;18:1227–31. 36660583 10.1016/j.radcr.2022.12.029 PMC9842795

[30] Kao DD, Fuu-Kou Y, Wang CS, Lehenbauer D, Zak S, Benscoter D, *et al*. Characteristics and outcomes of surgical treatment for bronchial anomalies. Laryngoscope. 2023;133:3334–40. 37159210 10.1002/lary.30737

[31] Bibas BJ, Cardoso PFG, Minamoto H, Pego-Fernandes PM. Surgery for intrathoracic tracheoesophageal and bronchoesophageal fistula. Annals of Translational Medicine. 2018;6:210. 30023373 10.21037/atm.2018.05.25 PMC6035972

[32] Kim HK, Choi YS, Kim K, Kim J, Shim YM. Long-term results of surgical treatment in benign bronchoesophageal fistula. J Thorac Cardiovasc Surg. 2007;134:411–4. 17662781 10.1016/j.jtcvs.2007.04.030

[33] Zacharias J, Genc O, Goldstraw P. Congenital tracheoesophageal fistulas presenting in adults: presentation of two cases and a synopsis of the literature. J Thorac Cardiovasc Surg. 2004;128:316–8. 15282472 10.1016/j.jtcvs.2003.12.046

[34] Anderson SA, Liu S, Galliani C, Weaver K. Isolated congenital bronchoesophageal fistula in a pediatric patient: case report. Am J Clin Pathol. 2020;154(Supplement 1):S43. 10.1093/ajcp/aqaa161.091.

[35] Ling Y, Sun B, Li J, Ma L, Li D, Yin G, *et al*. Endoscopic interventional therapies for tracheoesophageal fistulas in children: a systematic review. Front Pediatr. 2023;11:1121803. 36911034 10.3389/fped.2023.1121803 PMC9992425

[36] Sautin A, Marakhouski K, Pataleta A, Svirsky A, Averyn V. Treatment of isolated and recurrent tracheoesophageal fistula in children: a case series and literature review. World J Pediatr Surg. 2021;4: e000316. 36475238 10.1136/wjps-2021-000316 PMC9716785

[37] Holmquist A, Wendt M, Papatziamos G, Svensson J, Wester T, Burgos CM, *et al*. Endoscopic chemocauterization with trichloroacetic acid for congenital or recurrent tracheoesophageal fistula in children with esophageal atresia: experience from a tertiary center. J Pediatr Surg. 2023;59:678–83. 37978000 10.1016/j.jpedsurg.2023.10.049

[38] Slater BJ, Pimpalwar A, Wesson D, Olutoye O, Pimpalwar S. Esophageal stents in children: Bridge to surgical repair. Indian J Radiol Imaging. 2018;28:242–6. 30050250 10.4103/ijri.IJRI_313_17 PMC6038208

[39] Goussard P, Andronikou S, Morrison J, Van Wyk L, Mfingwana L, Janson JT. Management of children with tuberculous broncho-esophageal fistulae. Pediatr Pulmonol. 2020;55:1681–9. 32275811 10.1002/ppul.24775

[40] Debourdeau A, Gonzalez J-M, Dutau H, Benezech A, Barthet M. Endoscopic treatment of nonmalignant tracheoesophageal and bronchoesophageal fistula: results and prognostic factors for its success. Surg Endosc. 2018;33:549–56. 30014327 10.1007/s00464-018-6330-x

[41] Banciu C, Aprotosoaie A, Vancea D, Taban S, Guse C, Budu O, *et al*. A successful treatment of broncho-esophageal fistula with esophageal stenting using direct endoscopic visualization. Medicina. 2024;60:524. 38674170 10.3390/medicina60040524 PMC11052262

[42] Ishimaru T, Shinjo D, Fujiogi M, Michihata N, Morita K, Hayashi K, *et al*. Risk factors for postoperative anastomotic leakage after repair of esophageal atresia: a retrospective nationwide database study. Surg Today. 2023;53:1269–74. 37017869 10.1007/s00595-023-02682-0

[43] Chittmittrapap S, Spitz L, Kiely EM, Brereton RJ. Anastomotic leakage following surgery for esophageal atresia. J Pediatr Surg. 1992;27:29–32. 1552439 10.1016/0022-3468(92)90098-r

[44] Allin B, Knight M, Johnson P, Burge D. Outcomes at one-year post anastomosis from a national cohort of infants with oesophageal atresia. PLoS ONE. 2014;9: e106149. 25153838 10.1371/journal.pone.0106149 PMC4143357

